# Dairy food consumption is beneficially linked with iodine status in US children and adults: National Health and Nutrition Examination Surveys 2001–2018

**DOI:** 10.1017/S136898002300071X

**Published:** 2023-09

**Authors:** Yue Qin, Christopher J Cifelli, Sanjiv Agarwal, Victor L Fugoni

**Affiliations:** 1 Department of Nutrition Science, Purdue University, West Lafayette, IN 47906, USA; 2 National Dairy Council, 10255 West Higgins Road, Suite 900, Rosemont, IL 60018, USA; 3 NutriScience LLC, 901 Heatherwood Drive, East Norriton, PA 19403, USA; 4 Nutrition Impact, LLC, 9725 D Drive North, Battle Creek, MI 49014, USA

**Keywords:** National Health and Nutrition Examination Surveys, Urinary iodine concentration, Dairy foods, Iodine, Milk

## Abstract

**Objectives:**

The objective of this study was to determine the association between the consumption of dairy foods with urinary iodine concentration (UIC) and iodine deficiency risk in a nationally representative sample of the US population.

**Design, Setting and Participants:**

24-hour dietary recall data and laboratory data for UIC (μg/l) from subjects 2+ years old US population participating in National Health and Nutrition Examination Surveys 2001–2018 were used (*n* 26 838) for analyses after adjusting for demographic covariates. Significant associations were assessed at *P* < 0·05.

**Results:**

Mean intakes of total dairy were 2·21, 2·17 and 1·70 cup equivalents (cup eq) among those 2–8, 9–18 and 19+ years, respectively. Of the dairy components, intake of milk was highest followed by cheese and yogurt for all age groups. Total dairy intakes were positively associated with UIC among those 2–8 years (*β* = 29·9 ± 9·9 μg/l urine/cup eq dairy) and 9–18 years (*β* = 26·0 ± 4·8 μg/l urine/cup eq dairy) but not associated among those 19+ years. Total dairy intakes were associated with lowered risks (30 %, 21 % and 20 % for among 2–8, 9–18 and 19+ years, respectively) of being classified as iodine insufficient (UIC < 100 μg/l) or lowered risk (47 %, 30 % and 26 % among 2–8, 9–18 and 19+ years, respectively) of being classified as iodine severely deficient (UIC < 20 μg/l).

**Conclusions:**

The results indicate that dairy foods are beneficially associated with UIC and lowered iodine deficiency risk.

Iodine is necessary for the production of thyroid hormone and is essential for proper growth, development and energy homoeostasis regulation^(1)^. Iodine deficiency can lead to goiter or hypothyroidism as well as more severe outcomes such as implications for reproduction, fetal development and brain damage in children and is the leading cause of preventable neurological disorders in the world^(2,3)^. Iodine requirements for humans depend on age, gender, physiology and body weight^(4)^. Importantly, iodine requirements for pregnant and lactating women increase substantially due to iodine’s important functions in fetal neurocognitive developments^(4,5)^. While overt iodine deficiency is rare in higher income countries like the US, women of reproductive age are at high risk of inadequacy for iodine if they follow restrictive diets, such as those without dairy, egg, seafood or are limiting their use of iodised salt^(5)^. Given the importance of iodine for normal growth and cognitive development, decreases in iodine intake and inadequate iodine status could have negative impacts on the developing fetus and nursing child.

The use of iodine supplements is low in the US population^(6)^; therefore, the majority of iodine intake comes from food sources. Iodination of salt has been used in the USA to help reduce iodine deficiency^(1)^. However, the consumption of iodised salt has decreased in recent years due to dietary recommendations to reduce Na intake^(7,8)^, increased consumption of restaurant and processed food which tend to use non-iodised salt and the switch to numerous trendy non-iodised salts^(9)^. Dairy foods make significant contributions to the nutrient intakes of children and adults in the USA. For all Americans 2 years and older, milk is the number principal source of 3 of the 4 under-consumed nutrients of public health concern identified by the 2020–2025 Dietary Guidelines for Americans^(5)^: Ca, vitamin D and potassium^(10,11)^. Additionally, dairy represents a significant food source of iodine^(1,12–14)^, and milk and dairy foods contribute significantly to iodine intake in the USA and Europe^(15–19)^. Previous studies have shown that dairy foods contribute more than 70 % of iodine intake among children between 2 and 10 years and around 50 % in adults^(19,20)^ and has high bioavailability for absorption^(21)^. A recent systematic review of fifty-seven national surveys from European countries reported that milk and other dairy products provide 40–50 % of children’s iodine intake in Norway, UK, Germany and Spain and 34–53 % of adult intake in Finland, Ireland, Norway and UK^(22)^. Similarly, other studies found dairy intake was positively associated with iodine status among women of child-bearing age^(23–25)^. However, both the continued downward trend in milk consumption along with the emerging popularity of plant-based milk alternatives could lead to increased risk of iodine deficiency^(26)^.

Urinary iodine concentration (UIC) provides a sensitive estimate of dietary intake because most of (around 90 %) the iodine consumed is excreted via urine^(27)^. According to NHANES data, the US population is classified as adequate according to UIC data^(28)^. However, the UIC for the general population has declined since NHANES started collecting urinary iodine data in the 1970s^(29,30)^. Indeed, research has found that US women of reproductive age, as well as pregnant and lactating women, have inadequate iodine status^(23)^. Despite being a good source of iodine, there is a lack of research on the association between milk and dairy consumption and iodine intake, especially in different race and ethnic groups in the US population. Therefore, the objectives of the current study are to determine the association between the consumption of dairy foods with markers for iodine status and risk of inadequate intake of iodine using urine iodine concentration as a marker of intake, in a nationally representative sample of US population. We hypothesise that urine iodine status is positively associated with dairy intake for all age groups and the risk of iodine inadequacy is less with higher dairy intake.

## Methods

### Database and subjects

Dietary recall and laboratory data for UIC (µg/l) were obtained from those participating in the National Health and Nutrition Examination Surveys (NHANES) 2001–2018. NHANES is a continuous, nationally representative, cross-sectional survey of non-institutionalised, civilian US population conducted by the National Center for Health Statistics (NCHS) using a complex, multistage, probability sampling design and the data are released in two-year cycles. All participants provided written informed consent. This study was a secondary data analysis that lacked personal identifiers and therefore did not require further institutional review. A detailed description of the subject recruitment, survey design and data collection procedures is available online,^(31)^ and all data obtained for this study are publicly available at: http://www.cdc.gov/nchs/nhanes/.

The present analysis combined NHANES data cycles 2001–2018 using 24-hour dietary recall data from participants aged 2+ years, excluding pregnant and/or lactating females and those with zero calorie intake. Subjects were separated into three groups based on age: 2–8, 9–18 and 19+ years. Subsequently, these groups were also stratified by race-ethnicity as non-Hispanic White, non-Hispanic Black, Hispanic and Asian based responses from the demographic files (the delineation of these four ace/ethnicities are only available for 2011–2018 and thus these analyses are limited to these NHANES cycles).

### Dietary intake and iodine variables

Dietary intake data were obtained from interviewer-administered 24-h dietary recall (day 1) using USDA’s automated multiple-pass method in the Mobile Examination Center^(32)^. This 24-h recall data included a description and the amount of the individual foods and beverages consumed on the previous day (midnight to midnight) for each participant. Participants 12 years and older responded for themselves while a proxy representative responded for those under 6 years and for those 6–11 years a proxy representative was available to assist in recoding intakes. Complete descriptions of the dietary interview methods for NHANES are provided elsewhere^(31)^.

Dairy intakes (total, milk, cheese and yogurt) were determined linking the Food Products Equivalents Database (FPED)^(33)^ for each NHANES cycle with respective 24-h recalls. FPED provides food group composition of each food and beverage consumed and directly provides information on total dairy, milk, cheese and yogurt. Quartiles of each dairy intake variable were determined by age group and then by age/race-ethnicity group.

UIC were determined using inductively coupled plasma-MS for NHANES 2001–2004 and inductively coupled plasma dynamic reaction cell MS for NHANES 2005–2018 and were available for one-third of subjects from 2001–2018 and for those 3–5 years for 2015–2018 (see: https://wwwn.cdc.gov/Nchs/Nhanes/2017–2018/UIO_J.htm). Population groups were classified as with iodine insufficient levels (UIC < 100 μg/l) and iodine severely deficient levels (UIC < 20 μg/l) using the thresholds set by WHO^(3)^ and percentage of the population in these groups were determined by dairy intake levels by age and age/race-ethnicity groups.

### Statistics

SAS software (version 9.4, SAS Institute Inc) was used for all statistical analyses. Data were adjusted for the complex sample design of NHANES using appropriate survey weights, strata, primary sampling units and day one dietary sample weights. Least square means (LSM) and standard errors (se) were generated using regression analyses across levels of dairy intake (non-consumers and 4 quartiles of intake) after adjusting the data for age, gender, ethnicity for age groups and then age, gender and household poverty income ratio for analyses stratified by age/race-ethnicity. A *P*-value of < 0·05 was deemed significant to assess relationship of iodine variables with dairy intake variables (non-consumers and 4 quartiles of intake). Logistic regression was used to assess the OR of being classified as below the iodine insufficient level (UIC < 100 μg/l) and the severely iodine deficient level (UIC < 20 μg/l) for dairy consumption groups with non-consumers as the reference group by age groups; these analyses were adjusted for age, gender and race-ethnicity. When 95th percentile lower and upper confidence levels of the OR did not include 1·0, results were deemed significant. If sample size was so small that there were less than 15 events (i.e. UIC < 100 or < 20 μg/l) in the total population or if there are less than 5 subjects in any of the 5 intake groups results are not provided.

In addition, given UIC values are typically used for group assessment of iodine status and it is recommended that spot UIC values for individuals should be adjusted for creatinine, we re-ran analyses using UIC:creatinine ratio as the variable of interest. These analyses were limited to the total population of those 2–8, 9–18 and 19+ years. In the absence of specific cut-offs to use for defining iodine insufficient and iodine severely deficient levels using UIC:creatinine ratio, we used percentiles to the UIC:creatinine ratio to approximate these conditions (< 25th percentile and < 10 % percentile, respectively).

## Results

The mean intakes of total dairy, milk, cheese and yogurt in consumers were 2·21 ± 0·02, 1·58 ± 0·02, 0·78 ± 0·02 and 0·46 ± 0·01 cup eq, respectively, for age 2–8 years; 2·17 ± 0·03, 1·38 ± 0·02, 1·11 ± 0·02 and 0·48 ± 0·02 cup eq, respectively, for age 9–18 years and 1·70 ± 0·01, 0·95 ± 0·01, 1·09 ± 0·01 and 0·57 ± 0·01 cup eq, respectively, for age 19+ years. 1 cup eq is approximately 237 ml milk or yogurt and 1·5 ounce or 42·5 g natural cheese, Quartile intake cutoffs of total dairy, milk, cheese and yogurt quartiles are available in Supplementary Table 1

**Fig. 1:**
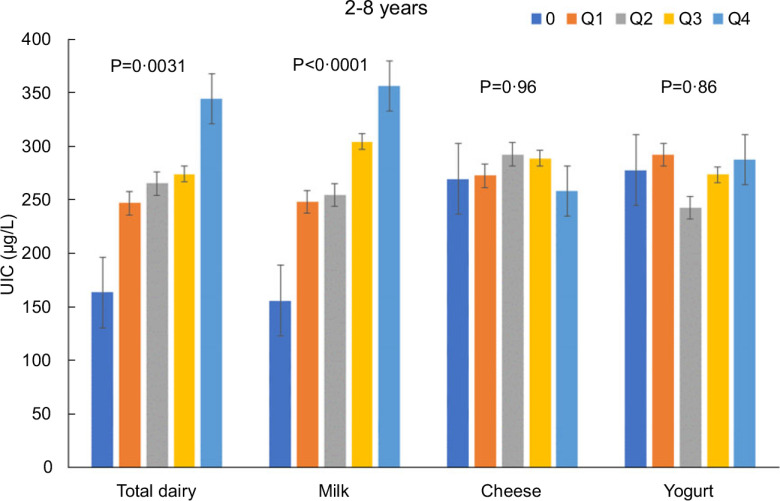
Association of increasing intake of dairy products with least square mean urinary iodine concentrations (UIC) among age group 2–8 years. NHANES 2001–2018 analysis. Data adjusted for age, gender and ethnicity. Actual values are available in supplemental Table 2

**Fig. 2:**
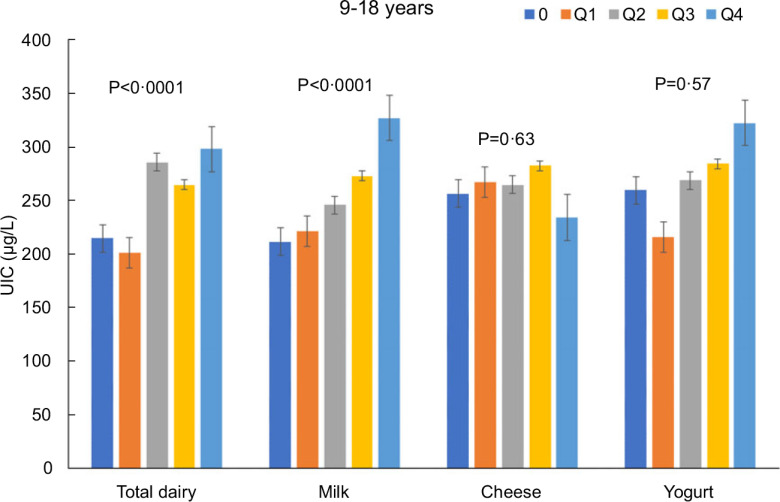
Association of increasing intake of dairy products with least square mean urinary iodine concentrations (UIC) among age group 9–18 years. NHANES 2001–2018 analysis. Data adjusted for age, gender and ethnicity. Actual values are available in supplemental Table 2

**Fig. 3:**
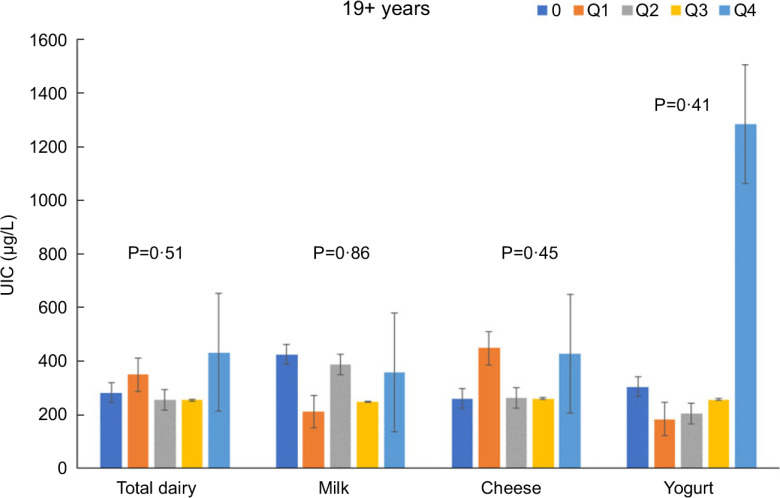
Association of increasing intake of dairy products with least square mean urinary iodine concentrations (UIC) among age group 19+ years. NHANES 2001–2018 analysis. Data adjusted for age, gender and ethnicity. Actual values are available in supplemental Table 2

Increasing intakes of total dairy were positively associated (*P* < 0·05) with UIC among those age 2–8 years (*β* = 29·9 ± 9·9 μg/l urine/cup eq dairy; Fig. 1) and 9–18 years (*β* = 26·0 ± 4·8 μg/l urine/cup eq dairy; Fig. 2) but not associated (*P* > 0·05) among those 19+ years (Fig. 3). Increasing intake of milk was also positively associated (*P* < 0·05) with UIC among those age 2–8 years (*β* = 39·8 ± 7·4 μg/l urine/cup eq milk; Fig. 1) and 9–18 years (*β* = 29·9 ± 6·3 μg/l urine/cup eq milk; Fig. 2) but not associated (*P* > 0·05) among those 19+ years (Fig. 3). Intakes of cheese and yogurt were not associated with UIC among any age group (Figs 1–3).

**Fig. 4:**
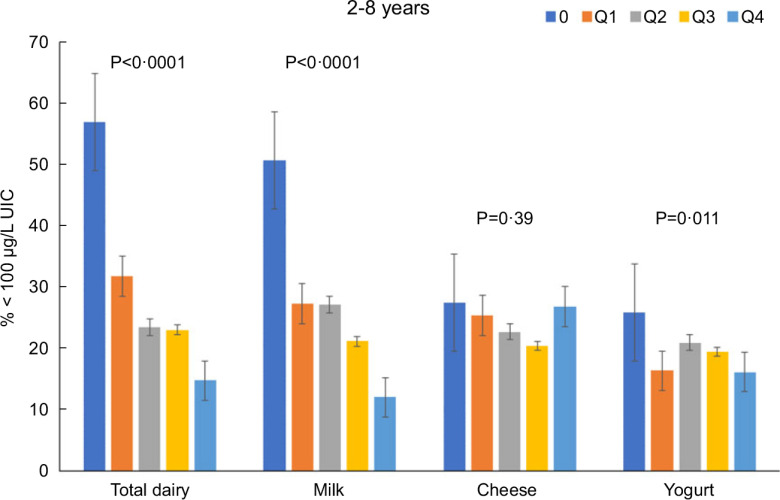
Association of increasing intake of dairy products with % population age 2–8 years classified as with iodine insufficient levels (% < 100 μg/l UIC). NHANES 2001–2018 analysis. Data adjusted for age, gender and ethnicity. Actual values are available in supplemental Table 2

**Fig. 5:**
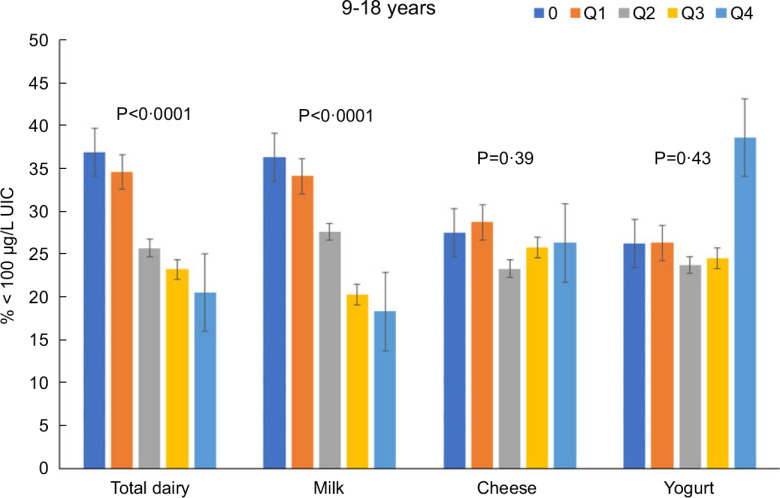
Association of increasing intake of dairy products with % population age 9–18 years classified as with iodine insufficient levels (% < 100 μg/l UIC). NHANES 2001–2018 analysis. Data adjusted for age, gender and ethnicity. Actual values are available in supplemental Table 2

**Fig. 6:**
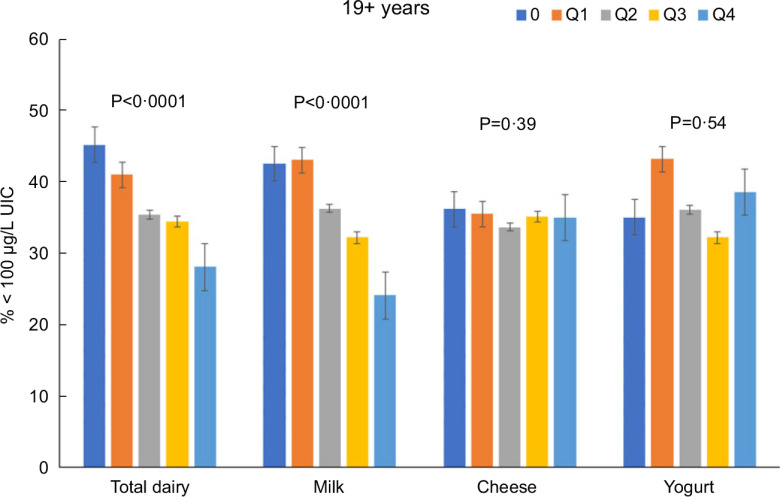
Association of increasing intake of dairy products with % population age 19+ years classified as with iodine insufficient levels (% < 100 μg/l UIC). NHANES 2001–2018 analysis. Data adjusted for age, gender and ethnicity. Actual values are available in supplemental Table 2

**Fig. 7:**
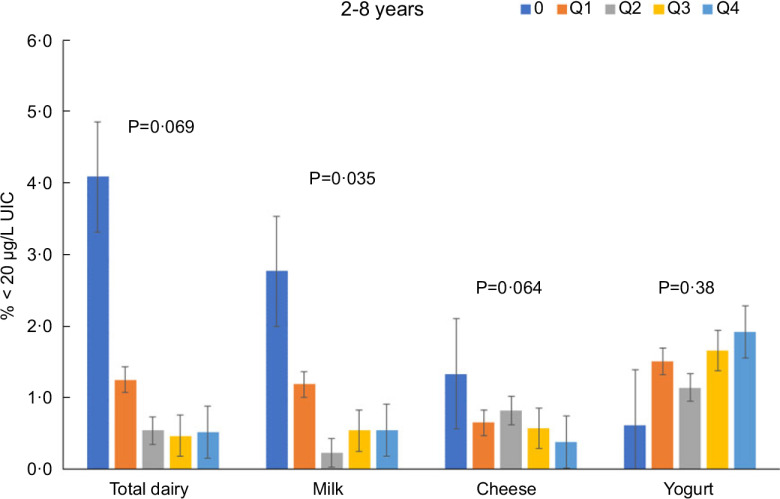
Association of increasing intake of dairy products with % population age 2–8 years classified as with iodine severely deficient levels (% < 20 μg/l UIC). NHANES 2001–2018 analysis. Data adjusted for age, gender and ethnicity. Actual values are available in supplemental Table 2

**Fig. 8:**
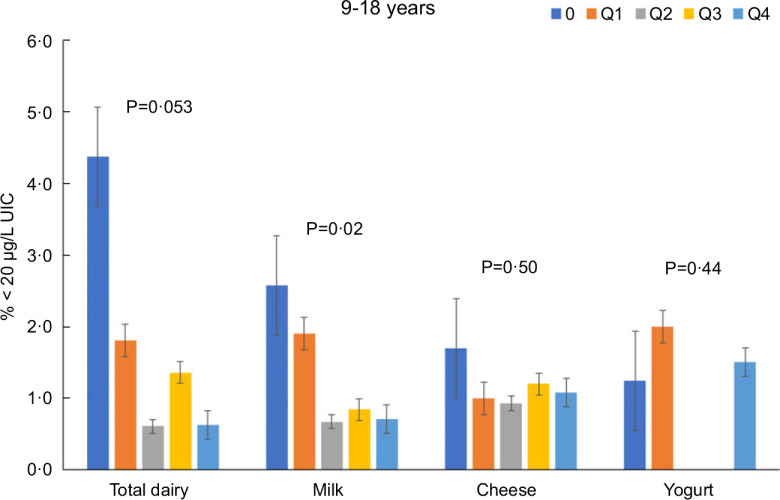
Association of increasing intake of dairy products with % population age 9–18 years classified as with iodine severely deficient levels (% < 20 μg/l UIC). NHANES 2001–2018 analysis. Data adjusted for age, gender and ethnicity. Actual values are available in supplemental Table 2

**Fig. 9:**
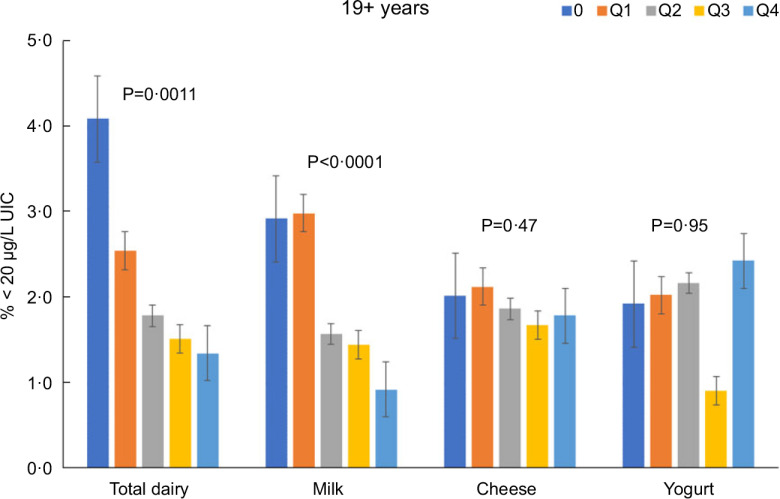
Association of increasing intake of dairy products with % population age 19+ years classified as with iodine severely deficient levels (% < 20 μg/l UIC). NHANES 2001–2018 analysis. Data adjusted for age, gender and ethnicity. Actual values are available in supplemental Table 2

Total dairy and milk intakes were inversely associated with the percentage of population that was classified as with iodine insufficient levels (UIC < 100 μg/l) among those age 2–8 years (Fig. 4), 9–18 years (Fig. 5) and 19+ years (Fig. 6). Yogurt intake was also inversely associated with percentage of those age 2–8 years classified as with iodine insufficient levels but not among ages 9–18 and 19+ years. Intake of cheese (for all ages) was not associated with the percentage of population that was classified as with iodine insufficient levels (Figs 4–6). Additionally, milk intake was inversely associated with percentage of the population that was classified as with iodine severely deficient levels (UIC < 20 μg/l) in those age 2–8 years, 9–18 years and 19+ years (Figs 7–9) albeit actual percentages were quite low (< 3 % of population). Intake of total dairy was also inversely associated with percentage of those age 19+ years classified as with iodine severely deficient levels (Fig. 9). Supplementary Table 2 provides data for each intake group stratified by age groups for UIC, % classified as with iodine insufficient levels and % classified as with iodine severely deficient levels.

Total dairy intake was inversely associated with OR (OR (95 confidence intervals)) of being classified as with iodine insufficient levels or iodine severely deficient levels among those 2–8 years (0·70 (0·61, 0·82) and 0·53 (0·28, 0·99), respectively), 9–18 years (0·79 (0·73, 0·85) and 0·70 (0·50, 0·98), respectively), and 19 + y (0·80 (0·77, 0·83) and 0·74 (0·65, 0·84), respectively). Milk intake was inversely associated with OR of being classified as with iodine insufficient levels or iodine severely deficient levels among those 2–8 years (0·67 (0·59, 0·77) and 0·49 (0·31, 0·79), respectively), 9–18 years (0·76 (0·71, 0·82) and 0·68 (0·50, 0·91)), respectively), and 19+ years (0·84 (0·80, 0·87) and 0·77 (0·66, 0·89), respectively).

While data for children 2–8 were unable to be analysed by ethnicity given the small sample sizes available, similar associations were found in analyses stratified by ethnicity in those 9–18 and 19+ years (data not presented).

Results of the relationship of dairy intake to iodine variables among those age 9–18 years by ethnicity are presented in Table 1. Total dairy intake was positively associated with UIC among non-Hispanic whites; total dairy, milk and cheese intake were positively associated with UIC among Asians. Total dairy and milk intakes were inversely associated with the percentage of population that was classified as with iodine insufficient levels among non-Hispanic whites and Asians. Similarly, cheese intake was inversely associated with the percentage of the population that is classified as with iodine insufficient levels among non-Hispanic whites. Total yogurt intake was inversely associated with the percentage of population that was classified as with iodine severely deficient levels among those Hispanic, non-Hispanic white and Asian. Total dairy and milk were also inversely associated with the percentage of the population that was classified as with iodine severely deficient levels in Asians. Similar results for those age 19+ years are presented in Table 2. Milk intake was positively associated UIC among Asians, while yogurt intake was positively associated UIC among non-Hispanic whites. Total dairy and milk intakes were inversely associated with the percentage of population that was classified as iodine insufficient among all ethnicities. Lastly, milk intake was inversely associated with the percentage of population that was classified as with iodine severely deficient levels among non-Hispanic whites and yogurt intake was inversely associated with the percentage of the population that was classified as with iodine severely deficient levels among non-Hispanic blacks. Supplementary Tables 3–6 provide data for each intake group stratified by race/ethnicity and age groups for UIC, % classified as with iodine insufficient levels and % classified as with iodine severely deficient levels.

**Table 1:** Association (quartile trends) of intake of total dairy, milk, yogurt and cheese with urinary iodine concentrations (UIC), percent of population classified as with iodine insufficient levels (% < 100 μg/l UIC) and iodine severely deficient levels (% < 20 μg/l UIC) among age 9–18 years

	Hispanic (*n* 626)			Non-Hispanic White (*n* 518)			Non-Hispanic Black (*n* 462)			Asian (*n* 210)		
	Beta	se	*P*	Beta	se	*P*	Beta	se	*P*	Beta	se	*P*
Urinary iodine concentrations (µg/l)												
Total Dairy	−2·85	5·85	0·6287	27·0	12·2	0·0316	−10·1	8·5	0·2426	89·1	20·5	0·0002
Milk	24·2	15·1	0·1150	16·4	18·6	0·3813	14·3	7·2	0·0573	48·4	16·6	0·0075
Cheese	−10·1	8·3	0·2296	−1·89	10·7	0·8602	−8·97	9·01	0·3265	48·8	17·3	0·0093
Yogurt	−1·50	14·47	0·9179	37·2	42·4	0·3841				25·4	24·8	0·3164
Percentage classified as with iodine insufficient levels (% < 100 μg/l UIC)												
Total Dairy	−0·31	1·75	0·8596	−9·17	2·13	0·0001	−0·37	1·81	0·8397	−7·16	3·38	0·0444
Milk	−3·22	1·82	0·0832	−7·19	1·90	0·0005	−2·14	1·74	0·2273	−10·0	3·1	0·0040
Cheese	0·88	1·39	0·5308	−3·90	1·75	0·0310	1·33	1·38	0·3409	−0·04	2·34	0·9875
Yogurt	−1·48	3·42	0·6681	−1·72	3·39	0·6154				−1·76	4·00	0·6639
Percentage classified as with iodine severely deficient levels (% < 20 μg/l UIC)												
Total Dairy	0·18	0·30	0·5454	−1·38	0·88	0·1234	−0·07	0·08	0·4075	−1·08	0·24	0·0001
Milk	−0·29	0·32	0·3798	−0·83	0·50	0·1042	−0·14	0·13	0·2986	−1·29	0·44	0·0069
Cheese	0·06	0·29	0·8250	−0·73	0·90	0·4230	0·32	0·32	0·3249	0·11	0·36	0·7584
Yogurt	−0·44	0·18	0·0196	−1·21	0·46	0·0122				−0·57	0·26	0·0366

Beta: regression coefficient; se: standard error of mean.

**Table 2:** Association (quartile trends) of intake of total dairy, milk, yogurt and cheese with urinary iodine concentrations (UIC), percent of population classified as with iodine insufficient levels (% < 100 μg/l UIC) and iodine severely deficient levels (% < 20 μg/l UIC) among age 19+ years

	Hispanic (*n* 1559)			Non-Hispanic White (*n* 2450)			Non-Hispanic Black (*n* 1465)			Asian (*n* 745)		
	Beta	se	*P*	Beta	se	*P*	Beta	se	*P*	Beta	se	*P*
Urinary iodine concentrations (µg/l)												
Total Dairy	44·2	32·8	0·1834	47·6	32·8	0·1524	−33·1	39·1	0·4010	24·8	15·0	0·1050
Milk	3·08	30·54	0·9201	46·5	30·0	0·1258	−37·3	32·3	0·2535	45·8	21·9	0·0412
Cheese	11·1	25·5	0·6646	−7·39	9·43	0·4364	−24·8	19·0	0·1972	3·33	14·73	0·8221
Yogurt	−2·53	21·17	0·9054	−32·3	12·5	0·0121	−3·53	28·19	0·9008	12·9	13·4	0·3384
Percentage classified as with iodine insufficient levels (% < 100 μg/l UIC)												
Total Dairy	−4·12	1·35	0·0034	−3·22	1·12	0·0057	−4·58	1·08	0·0001	−5·68	1·90	0·0041
Milk	−3·02	1·18	0·0132	−4·04	0·96	0·0001	−2·93	1·11	0·0111	−4·89	1·80	0·0089
Cheese	−2·17	1·21	0·0775	−0·87	0·98	0·3761	−1·58	0·87	0·0759	−1·23	1·39	0·3812
Yogurt	0·39	1·94	0·8433	2·83	1·50	0·0650	−1·15	2·93	0·6967	−2·03	2·37	0·3947
Percentage classified as with iodine severely deficient levels (% < 20 μg/l UIC)												
Total Dairy	0·37	0·39	0·3537	−0·69	0·47	0·1454	−0·53	0·49	0·2890	−1·04	0·79	0·1929
Milk	−0·43	0·67	0·5218	−1·09	0·27	0·0001	0·34	0·36	0·3580	−1·01	0·57	0·0827
Cheese	0·59	0·52	0·2632	−0·28	0·32	0·3877	−0·67	0·33	0·0501	−0·70	0·85	0·4177
Yogurt	0·13	0·73	0·8547	0·31	0·64	0·6286	−0·75	0·23	0·0018	2·08	1·79	0·2490

Beta: regression coefficient; se: standard error of mean.

Regarding analyses using UIC:creatinine ratio as variable of interest, we found results were consistent with those found using UIC values (online Supplementary Table 7). In particular, total dairy and milk intakes were inversely associated with the percentage of population that was classified as iodine insufficient (defined as < 25th percentile of UIC:creatinine ratio) and severely iodine deficient (defined as < 10th percentile of UIC:creatinine ratio) in each of the age groups evaluated.

## Discussion

The current study indicated that dairy intake was positively associated with iodine status and inversely associated with risk of being classified as with iodine insufficient levels and severely deficient levels among the age groups assessed.

Specifically, total dairy and milk intake was positively associated with UIC, the recommended method to assess iodine adequacy^(27)^, among children, inversely associated with percentage of iodine insufficient among all ages and inversely associated with percentage of iodine severely deficient among adults 19 years and older. Similar links were also found across the different racial/ethnic groups examined. Recent studies have suggested that iodine deficiency is reappearing in some European nations that had previously been sufficient^(34)^. The Dietary Guidelines Advisory Committee found a similar trend among some populations in the USA^(28)^. Our findings show that higher dairy intakes are linked with improved iodine status; however, mean intakes of dairy across all the age groups in this study were below recommendations. Therefore, meeting dairy recommendations likely represents one way to help ensure adequate iodine intake.

Iodine is an important trace element. Low iodine intake is a public health concern, particularly among pregnant and lactating women, because of the consequences of iodine deficiency. Besides the fatigue, mental slowing, weight gain and depression that is characteristic of iodine deficiency in adults^(4,28,35)^, it is also one of the leading causes of preventable intellectual disability worldwide. Public health recommendations to limit salt intake coupled with ever-changing trends in dietary habits have resulted in a general shift away from iodised salt consumption. Consumer surveys have shown that only 53 % of table salt sold in the USA is iodised and table salt accounts for only < 30 % of total daily intake of salt^(9,35)^. In additional, some multivitamin/multimineral supplements marketed to pregnant women or planning to become pregnant do not contain iodine. Therefore, given current dietary patterns and trajectories, iodine could reemerge as a nutrient of public health concern in future dietary guidance especially among pregnant and lactating women.

Previous research has found similar associations between dairy intake and iodine status. One study found recent dairy intake was positively associated with UIC among US children 6–12 years^(36)^, which agree with the findings from the current analysis for US children. On the other hand, Lee et al. found positive association between dairy consumption and UIC among US adults 20 years and older^(37)^, yet the current analysis did not find this association among US adults 19 years and older. This might be due to the difference in population included, covariates adjusted and survey cycles used. Previous studies excluding pregnant and lactating women did not focus on the link between dairy intake and iodine insufficiency/deficiency or stratify by race ethnicity. Research has found race/ethnicity was significantly associated with UIC^(38)^ and dairy consumption among women of reproductive age^(29)^, while similar associations were found in the current analysis among children 9–18 years and adults 19 years or older.

Results from the current analysis indicate dairy, specifically milk, represents an important source of iodine intake from diet and could potentially play a crucial role in preventing and combating iodine deficiency. Milk is an excellent source of iodine and contains 350–425 μg iodine per liter milk^(39,40)^. The iodine content of milk can be variable because of different farming practices including cattle feed and sanitation^(39,41)^. Iodophor disinfectants, used for preparations and treatments for sanitation, or feed that has been supplemented with iodine are two possible ways that the iodine content of milk can be increased^(39)^. Processing methods will also impact the iodine content in milk. The presence of goitrogens can also impact/reduce iodine intake or uptake. Furthermore, some studies have shown that dairy products produced from cows feeding on pasture tend to have higher iodine concentrations although geographic location will directly impact the amount of iodine in the pasture^(39,42)^. In a recent USDA analysis of 96 milk samples, the authors explained the large sample-to-sample variation in iodine content was largely due to iodine supplementation and/or use of iodophors during milking^(39)^. In the USA, dairy products contribute to nearly half of the total iodine intake from foods^(43)^. Indeed, the results of the present analysis showed that milk intake was positively associated with iodine status in this cohort. We did not analyse different milk types by the fat contents; however, a recent study of US milk samples indicated that mean iodine content did not differ among milk fat levels^(39)^. In contrast, results from the current analysis did not find strong links between markers of iodine status and intake of cheese and yogurt, partly due to small sample sizes from low consumption. Future analyses are warranted to understand the iodine content of these dairy products more thoroughly. Along the same lines, research is needed to better understand the iodine content of all foods so that dietary pattern studies can be conducted which will directly inform future food-based dietary guidance.

The current study is one of the few to examine the link between dairy consumption and population iodine status in children and adults from different ethnic groups. Herrick et al. reported median UIC highest among non-Hispanic whites, followed by all Hispanics, non-Hispanic blacks and lastly Asians among women of reproductive age^(29)^. The present analysis showed that the highest UIC observed was among non-Hispanic whites, followed by non-Hispanic blacks, Hispanics and Asians for children 9 to 18 years and highest UIC among non-Hispanic blacks, followed by non-Hispanic whites, Hispanics and Asians for adults 19 years and older. The differences between the two studies were likely due to the different population included and survey cycles used for the analysis. Nonetheless, despite the differences, both studies reported differences in iodine status across racial/ethnic groups highlighting an incidence of possible nutrition inequity. Further to that point, the current study observed that largest regression coefficient for the increase in UIC was found for total dairy, milk and cheese in Asians 9–18 years. Regarding dairy, we also observed differences in milk and total dairy by race and ethnic group with non-Hispanic blacks consuming the least amount of milk. While there are many factors that influence milk intake, perceived or real lactose intolerance is a principal factor influencing milk consumption in these groups. Other factors, such as misconceptions of health effects of dairy (concerns for high energy content and cholesterol), eating environment, peer influence, personal dietary behaviors (meal skipping or availability of dairy products) and cultural influenced dietary habits (Asian diets do not incorporate as much dairy products as western diets), all contributes to disparities of dairy intake across race ethnicity groups and especially the low intake among certain groups^(44)^. Given the accessibility and affordability of milk, increasing the awareness of lactose-free milks among these ethnic groups to help increase dairy intake to recommended levels could have a profound impact on iodine status.

Milk makes a meaningful contribution to the RDA for iodine on a population-wide basis in the USA. However, there is an increasing awareness of the environmental impacts of the food system^(5)^. Indeed, some people have moved to or recommended more restrictive diets that either eliminate or reduce not only dairy but also other animal sources of iodine like eggs. In part, this has contributed to a growing interest in plant-based foods including plant-based beverages. However, most plant-based alternative drinks are not fortified with iodine, which could lead to decreased intakes and levels. Two studies examining the iodine content of milk alternatives have shown that the iodine content of milk alternatives was significantly less as compared with cow’s milk^(45,46)^. Soymilk and soya products also contain substances such as flavonoids which are goitrogenic and affect thyroid peroxidase enzyme which could contribute to decreased UIC^(47)^. It is important to note that the impact of the consumption of milk alternatives on iodine intake and status will depend on the overall quality of the diet. Indeed, individuals who chose alternatives will need to increase iodine intake from other food sources. Nonetheless, existing research indicates that milk alternatives are not equivalent to milk and might lead to unintended health consequences. The lack of nutrition equivalence, including for iodine, is one reason why health professional organisations and the Dietary Guidelines for Americans do not recommend alternative milks, especially for children^(5,8,48)^.

There are limitations for the present study. The analyses rely on self-reported intakes of diet which could be subject to error and bias. However, all self-reported dietary assessments suffer from some extent of errors and misreporting and have the most impact on total energy intake^(49)^. The 24-hour dietary recall has been one of the most validated tool compared with other dietary assessment tools and provide valuable information with adequate accuracy for intake of a specific nutrient^(50)^. Therefore, using data collected from 24-hour dietary recall was appropriate considering the research question of the current study. Additionally, salt intake was not examined or controlled. However, it is unlikely that salt intake would have a direct impact on the current findings given the low usage of iodised salt. Pregnant and lactating women were not included. This decision was made because of different iodine recommendations and potentially higher intake of iodine from supplements associated with this population, which could heavily skew the results. This was a common practice in previous analyses. Data on those age 2–8 years could not be included for analysis with stratifications by race/ethnicity due to small sample size and missing data. The use of a single spot UIC sample may not provide information on long-term iodine status of an individual. Additionally, it is only appropriate for population assessment for an individual, the iodine-to-creatinine ratio, or a 24-h urine sample, is preferable. While we did run analyses of iodine-to-creatinine ratio, we are unaware of specific cut-offs to use for defining iodine insufficient and severely iodine deficient levels so we used certain percentiles to the urinary iodine: creatinine ratio to approximate these conditions (< 25th percentile and < 10 % percentile, respectively). Lastly, over the duration of our analyses time frame, there was a change in measurement technique for urinary iodine, and we are unaware of any information regarding the alignment of the methods used.

## Conclusions

The current findings provide evidence to support the dietary recommendations. Specifically, the importance of ensuring adequate dairy intake could aid in preventing a potential upcoming public health crisis and serve as helpful tools to tackling existing deficiencies. Future research is needed to better understand relationship of dairy consumption and iodine markers for population of all ages as well as long-term impact of dairy consumption on iodine status.

## Supplementary material

For supplementary material accompanying this paper visit https://doi.org/10.1017/S136898002300071X. click here to view supplementary material
